# OP50 NICE Listens: Engaging The Public On How Environmental Sustainability Should Be Considered In Health Technology Assessment

**DOI:** 10.1017/S0266462324001120

**Published:** 2025-01-07

**Authors:** Alice Murray, Koonal Shah

## Abstract

**Introduction:**

Involving the public is essential to building trust in health technology assessment (HTA) organizations. The National Institute for Health and Care Excellence (NICE) runs a deliberative public engagement program, NICE Listens. It was used to explore informed public opinion on how environmental sustainability should be taken into account in HTA.

**Methods:**

Twenty-three general public participants from across England took part in three iterative online workshops (each lasting two or three hours, held three weeks apart in 2022). The workshops included trade-off exercises, role-play, group discussion, and video clips from interviews with sustainable healthcare experts.

**Results:**

Strong support was found for NICE taking action to make healthcare more environmentally sustainable. Support increased as participants learned that sustainable healthcare offers co-benefits, such as reduced burden on the National Health Service through better self-management of conditions. Participants did not want health outcomes to be compromised in pursuit of sustainability. We identified some circumstances where they found it acceptable to consider the environmental impact of interventions in decision-making: when effective treatments already exist; when the condition is not severe; when the alternative is equally cost effective; and when greener options are marginally higher in cost but as clinically effective as the alternative.

**Conclusions:**

The findings demonstrate that environmental sustainability is clearly considered a relevant element of value. They also offer insight into how the environmental impact of health interventions should be considered in HTA. Further research should focus on methods for consistent measurement of the environmental impacts of health interventions and the incorporation of those impacts into decision-making.

